# Complexity, Uncertainty, and Entropy: Applications to Food Sensory Perception and Other Complex Phenomena

**DOI:** 10.3390/e27020191

**Published:** 2025-02-13

**Authors:** Luka Sturtewagen, Harald van Mil, Erik van der Linden

**Affiliations:** 1 Laboratory of Physics and Physical Chemistry of Foods, Wageningen University and Research Center, Bornse Weilanden 9 (Building 118), 6708 WG Wageningen, The Netherlands; luka.sturtewagen@gmail.com; 2 Mathematical Institute, Leiden University, 2333 ZA Leiden, The Netherlands; h.g.j.van.mil@math.leidenuniv.nl

**Keywords:** entropy, complexity, sensory, information, diversity, ecology

## Abstract

Complexity has been studied in various areas of science, such as ecology and sensory science. One important aspect is the quantification of the complexity of a system. There exist a multitude of different approaches. One approach relates complexity to information, with its measure introduced by Shannon. This is equal to the negative value of entropy, or uncertainty. In this review, we discuss the different approaches that are used to quantify and measure complexity in the realm of food sensory perception. We address how the food sensory field could benefit from an approach that is based on an information-theoretical footing, to allow for one quantitative measure, thus improving comparison and reproducibility among different laboratories. Reversely, the review is intended to inspire the physics community to explore complex phenomena, such as sensory perception, in terms of a general information-theoretical measure, such as entropy and other fields.

## 1. Introduction

The concept of complexity may enable the crossing of boundaries between traditional scientific disciplines by identifying according measures to allow for the analysis and interpretation of high-dimensional phenomena. The field of food sensory perception is such a field that crosses different disciplines, from chemistry and physics to psychology, and, indeed, the concept of complexity is an important concept in this field.

In defining the concept of complexity in relation to food sensory perception, Köster et al. [1] stated that the concept of complexity is either defined a priori on the basis of information-theoretical considerations, or measured, with the help of rating scales or paired comparison, by a panel.

Commenting at this point on the use of the term “information-theoretical”, it should be noted that if something is more complex, the amount of information used to describe it will be larger. In this sense, one may equate complexity with information. The quantification of the term “information” is based on the fact that when a certain amount of information on a system has become observable, it is this information that is not hidden anymore. Before such an observation became possible, this information was hidden within the system. Because that amount of information was hidden, it added to the uncertainty of describing the system. In other words, the uncertainty that is related to describing a system equals the negative value of the information available in the system. Now, the uncertainty in describing a system is determined by the number of possibilities that a system has internally. In fact, Shannon argued that an expression for this uncertainty equals the expression for the entropy of a system, analogous to the entropy as a function of the number of possible states that a system can attain in physical descriptions of a system. It is noted that the thermodynamic concept of entropy is well defined for physical systems, and relates to the number of possible states for such a system. For “a person consuming food”, the principal number of possible states, and a thermodynamic interpretation of the entropy and information of “a person consuming food”, are not available. Shannon strictly applied the information measure to the problem of efficient and errorless communication between a sender and a receiver, and explicitly expressed his concerns towards the possibility of erroneously extrapolating his arguments to situations outside of the communication area. Nonetheless, discarding this warning, many people have utilised a direct extrapolation of the Shannon information measure in other areas, including the area of food sensory science, and we will use this extrapolation as well. Some more background information on information and entropy can be found in the introduction by Sturtewagen et al. [2]. Using the concept of the number of possible states of a system, and the above-explained Shannon information measure, one can use the observed number of bits in an observed pattern as a measure for the information. Such an approach, and its relation to complexity, has been conducted in many areas of science, in different model systems and interpretation schemes. The popularity of using the Shannon information measure is partially due to its mathematical properties, which also give this measure a meaning in a practical sense. An example of how this information-theoretical measure was recently applied to sensory time–intensity evaluations during chewing of a food, in relation to the moment of swallowing, can be found in Sturtewagen et al. [2]. It was found that using this measure, the information obtained from sensory perception during chewing until the moment of swallowing showed one master curve for all participants in the study, when plotted versus individually normalised chewing time.

Apart from the information-theoretical considerations in the above paragraph, it is important to realise that the term “information” is related to the context. Namely, the information coming to a perceiving individual partially comes from the information available in a given context, e.g., the surroundings of the individual. This contextual information is processed in the individual together with information coming directly from the food stimuli. The processing of this food-related information with the contextual information may lead to interference, and the information, after being processed, contains a contextual part. If complexity is seen as a perceived signal or perceived indicator of important food sensory markers, it would be expedient to define this context in identifying the complexity of the perception.

Apart from using the above quantification of complexity in an information-theoretic way, other routes are used. The most often-used alternative is one according to a much-cited paper by Berlyne [3], in which something becomes more complex when there are (1) more distinguishable elements, (2) when there is more dissimilarity between the elements, and (3) when the elements cannot be grouped into a single class or unit. The study by Berlyne was performed on visual stimuli, which are just one of the types of stimuli that are relevant in food sensory science. The multifaceted description is hypothesised to link the stimulus’ complexity to its arousal potential and hedonic value [3,4,5] by following an “inverse U-shaped-curve”, which is often being used throughout food sensory science.

We note that (1) suggests a linear relation between complexity/information and the number of elements, N, while the expression of information based on Shannon’s arguments contains a logarithmic term instead of a linear term. Apart from these two routes of quantifying complexity, there exist multiple other, albeit similar, connotations and measures for complexity. This makes interpretation and interlaboratory comparisons of the measure ambiguous. When the complexity of a stimulus, such as sensory perception of a (model) food product or beverage, is studied, there are different views on what a complex stimulus entails [6]. For example, in a study on the perceived complexity of wine, Parr et al. [7] concluded that wine experts and wine novices had different interpretations of the term “perceived complexity”. To make matters worse, perceived complexity and sensory complexity are often used interchangeably [8]. In food sensory science, complexity has been studied mostly with regard to flavour and aroma perception. The complexity of flavours has been studied both in foods, such as yoghurts [9], chocolate [10], and various foods with added spices [11], and in drinks, such as orange juices [12], beers [13] and wine [14]. Here, the scale of complexity is related to the stimulus. Much of the perceived complexity or the sensation of complexity in flavour and aroma research is based on research in fragrance [15,16] and scent [17]. Different measures of complexity have been related to key sensory variables, such as sensory-specific satiety [18], the expected satiating capacity of food products [6], arousal potential, liking [12,13,19,20,21,22,23,24,25], or the food-neophobic scale [11]. Many studies show a linear relationship between perceived complexity and the number of stimuli, number of compounds [9,11,18,23,24], difficulty of describing or identifying the stimulus [9,11,23], or inverse of simplicity [10,15,20,21,22]. Such linear relations might point to an additive property of the complexity measure used. However, some authors do not find, or expect, a linear relationship when interactions between components are involved [8,26].

The different approaches in the literature to quantifying complexity and its use for sensory perception, in relation to the measure of complexity as is known from the physics field, will be the main focus of this review paper. We will refrain from giving experimental details for the examples addressed or calculating other possible measures of complexity for specific experiments. We also refrain from discussing relations between instrumental and sensory measures. This topic is beyond the scope of the current review. Usually, instrumental measures also only represent a pale reflection of sensory reality. This is why sensory panels are a very important tool in assessing the quality of food and new developments thereof, and why we have focused on sensory measurements alone.

Taking Berlyne’s view as a starting point, we first review some of the food sensory literature and identify the type of observation and context on which a measure of complexity is based. Then, we explore analogies with similar measures that are utilised in the field of ecology, like species richness and diversity. New results developed in the ecological and socio-economic contexts that deal with similar complications as in the sensory area are mapped to the context of food sensory science, in order to relate the different classes of complexity observed. After this, we treat, in more depth, the specific information transfer between food and individuals in relation to complexity. In the subsequent section, we treat sensory experiments performed to extract complexity and information by means of identifying perceived dissimilarities. In fact, this resembles measuring the uncertainty and entropy more directly, i.e., the negative of information. We end with a discussion of future directions.

## 2. Complexity in Food Sensory Science

The concept and measures of complexity have relevance at different levels in food sensory science, as discussed in [27]. In order to structure the discussion below, we need a generic measure for complexity that is rich enough, with links to empirical science and statistics, and can at least partially accommodate some of the critiques of existing measures. As mentioned in the introduction, one such a candidate is information entropy, by means of assuming the validity of generalising the information entropy in Shannon’s theory of communication [28]. The most common interpretation of entropy is uncertainty, and as such, the term has been introduced as a measure for surprise in food sensory science, or as a measure for biodiversity in ecology. While applications of information entropy within complexity science are manifold, its application within the behavioural sciences is not without criticism. Luce [29] challenges the usefulness of information theory in psychology due to its inability to allow for any structural relations between stimuli. Luce refers frequently to papers by Laming [30,31], and reiterates Laming’s criticism that string size (the maximum number of possibilities for a parameter), which forms an important element within the context of information theory to deal with redundancies, is seldom considered in psychological experiments. Laming, however, concludes in his paper [30] that although, in most cases, information theory is not very useful, a notable exception might be found in the investigation of complex systems when a detailed description of the underlying mechanism is not available. In this paper, we will use Shannon information entropy, and the properties of complexity, as discussed by Berlyne, as concepts to structure our discussion of the use of a measure of complexity and its interpretations in food sensory science. This approach allows for an investigation of the relations between the different measures of complexity that are used in the food sensory literature.

### 2.1. Complexities of Different Sorts: Stimulus, Sensory, Perceived, and Descriptive Complexity

To allow for a more structured classification of the complexities used, we will first discuss four basic contexts in which the concept or a measure of complexity is used in food sensory science, i.e., stimulus, sensory, perceived, and descriptive complexity. We note that representations are numerical (both discrete (counts) and continuous (slider, Visual Analogue Scale (VAS)), or categorical (x-level Likert scale).

We start with the stimulus complexity (numeric) of a food/sample, which, in most cases, is related to the number of ingredients, like flavour, aroma, textures or visual cues, but in some cases, is related to the heterogeneity of the food. As such, stimulus complexity might correlate food properties with behavioural properties like acceptance.

Sensory complexity (numeric), like stimulus complexity, is related to the number of ingredients or attributes that can be distinguished by the study subjects or panel. One might view this as a subjective representation of the stimulus complexity, and therefore a property of the test subject.

Perceived complexity (numeric or categorical) is measured on a numerical scale using the Visual Analogue Scale (VAS), where the subject needs to score the perceived level of complexity between the anchors “not at all” and “very much”; or in a categorical version, like an x-level Likert scale, between the anchors “not at all complex” and “extremely complex”. This directly relates a property of the test subject to a measure of complexity.

As a fourth context, we have descriptive complexity, which measures the difficulty of describing a sensation (numerical and categorical). Here, the length of the description of the sensation can be a numerical measure for the complexity of the sample, or an x-level Likert scale, with anchors between easy and very difficult. This is a subjective measure, and the property of the subject serves as the observed variable for the latent variable complexity.

Some authors combine the above different classes of complexity into one measure. An example of this is in a combination of sensory complexity, compound perceived complexity, and descriptive complexity [9].

### 2.2. Food Sensory Studies and Different Sorts of Complexity

Olabi et al. [11] compared two versions of the same foods, in neophilic and neophobic subjects, with one sample having a more complex flavour. The complexity of the flavour was controlled by adding more flavours, in which case we can talk about stimulus complexity. Subjects scored the samples based on perceived complexity, acceptability, familiarity, and expectation. The perceived complexity was measured using the 2-AFC (Alternative Force Choice) test. The 2-AFC test is a two-level sensory test used to select the most complex sample, followed by a three-level test to test how sure the subject is regarding the selection. Six out of eight flavoured samples were considered to be more complex, and the acceptability of the more complex samples was higher for the neophilic group.

Weijzen et al. [18] studied the effects of intensity and complexity on sensory-specific satiety and food acceptance of various chicken soups and snacks, such as chocolate bars with incorporated nuts, with different flavours and textures. The set of samples was selected based on the samples’ perceived complexity and perceived intensity on a nine-level Likert scale. Participants assessed the complexity of the products by asking the panellists directly how complex the product was (perceived complexity, Likert scale), how many different ingredients it contained (sensory complexity, Likert scale), and how difficult it was to describe it (descriptive complexity). From their results, the authors concluded that perceived complexity was strongly correlated with sensory complexity and that descriptive complexity was inversely related to familiarity. They concluded that intensity increases, and perceived complexity decreases, sensory-specific satiety.

Ruijschop et al. [9] investigated the influence of complexity on satiation and food intake. Their samples consisted of two types of strawberry-aromatized sweetened yoghurts: one with a single-component strawberry aroma, and one with a multi-component strawberry aroma (stimulus complexity). They assessed the complexity by asking the panellists about the difficulty of describing the taste (descriptive complexity, VAS), the perceived number of ingredients (sensory complexity, VAS), and the perceived level of complexity (perceived complexity, VAS). Their study indicated that the more complex aroma had higher perceived complexity scores, but had similar intensity to the single-component aroma. Pleasantness, compared with the single-component strawberry aroma, showed enhanced satiation.

Paulsen et al. [32] indicated that for the composite properties of meals, harmony and complexity can be used to describe a meal or a dish, such as fish combined with a sauce, but it is difficult to relate specific sensory attributes to such descriptions. They defined complexity as the sensation of many tastes and flavours which is conceptually close to sensory complexity, but is a measure like perceived complexity (intensity of complexity). This study indicated that bitter, acid, and salty sauces significantly affected perceived complexity.

Lévy et al. [12] studied the perceived complexity of orange juices to which they added different flavour extracts. Perceived complexity was considered as a measure of a combination of scores on a nine-point scale for three attribute dimensions, using the anchors of simple–elaborateness, straight–confusion, and homogeneity–scattered. The optimal level of complexity was found to be related to the most-liked product. The differences between the scores of the complexity attributes for a product, and the scores of those attributes for the most-liked product, was defined as the perceived complexity. The difference divided by the complexity of the most-liked product led to a scaled relative perceived complexity. Then, the sums of the scores were used as a measure of the perceived complexity, grouped around 0, representing the optimal complexity. The first two principal components in a principal component analysis indicated an almost linear relation with the simple–elaborateness and homogeneity–scattered attributes. The straight–confusion attribute correlated more with the second principal component only. From this, we can conclude that their use of the term “perceived complexity” belongs to the perceived complexity class. The study concluded that after exposing the subjects to samples with higher complexity, their optimal complexity perception was shifted upwards, without effects on low complexity.

Soerensen et al. [10] wanted to predict long-term consumer liking of chocolate. They defined perceived complexity on a 15 cm line scale with nine verbal anchors, ranging from extremely simple to extremely complex. Although sensory complexity was discussed by them, it was not measured, but described as the number of perceived flavours, and the number of perceived flavours was not recorded. They pointed out that this would have been a hard task, as there have been more than 800 flavour compounds found in chocolate. At the same time, to complicate matters even more, these compounds are not released at the same rate and the same time. Their release is dependent on eating behaviour and oral processing. This causes flavour perception to be a dynamic phenomenon. The problem becomes even more difficult because of interpersonal differences in perception and differences in previous experiences between panellists. Jellinek and Koster [15] discussed this problem in terms of the many receptors involved at different times during perception, and determined the problem to lie in the area of physiological complexity.

There has been a lot of confusion between the complexity of a stimulus and the complexity of a sensation [8]. The number of different sensations is not always consistent with the number of stimulus components. The perceived complexity of a sensation does not always correlate very well with the number of compounds [15] or stimulus complexity. A single compound may be perceived as more complex than a multi-compound mixture. Therefore, the number of ingredients alone is not enough of an indication of what the perceived complexity of a product will turn out to be.

The complexity of texture perception was studied by Larsen et al. [33] and Marcano et al. [6]. Larsen et al. [34] defined “textural complexity”, or sensory complexity according to our classification, to be proportional to the number of texture attributes sensed during mastication of a food product, from the first bite through to the point of swallowing. The number of texture attributes is established by asking the panel to name all the attributes they perceive while consuming the sample. For the sensory assessment, they used qualitative descriptive analysis (QDA), texture profiling (TP), and temporal dominance of sensations (TDS). For their study, they used model foods with layers of different mechanical properties created by incorporating various gelled discs and seeds. In later studies, they analysed the food bolus at different stages of the chewing process [35], conducted satiation experiments [33], and assessed mechanical properties using a texture analyser [36]. They saw a “clear upward trend” in the number of unique descriptors for the different samples during the QDA. From this, they concluded that their samples had different perceived texture complexities. They concluded from the TDS that for more texturally complex samples, there were more texture attribute changes, and more texture attributes were perceived as dominant.

Marcano et al. [6] studied the texture perception of different cheese pies with visible particles added to the recipe. They hypothesised that these particles would increase the perceived complexity of the texture and the expected satiety. During their experiments, consumers were asked to rate the perceived complexity on a nine-point scale, from not complex at all to very complex. Moreover, panellists were asked to indicate which characteristics they included in their assessment of the perceived complexity of the pies. Using a flash profiling set-up, panellists were asked to make a list of attributes they used to describe the differences between the different cheese pie samples. Then, the panellists rated the samples based on a 0–10 score of their selected attributes. A high complexity coincided with a lack of homogeneity, as indicated by the use of the terms “heterogeneity” and “texture”. The authors concluded that a higher perceived complexity coincided with a higher satiation expectation. The most frequently mentioned terms for the complexity were related to the particle characteristics and to the texture (it is not clear what is meant by “texture”, the generic term used).

Reverdy et al. [24] studied the effects of the intensity and complexity of different food products on children, and investigated how sensory education had an effect on their food preferences. They varied the ingredients of the products and asked the children to indicate which sample had more tastes, and which sample was more surprising, to assess the complexity of the different products.

The complexity of visual perception was studied by Mielby et al. [21,22]. They assessed the preference of adolescents for pictures of fruits and vegetables mixed in different ratios and cut in different patterns. They studied how the perceived complexity of the different mixes influenced preference for the mixes. The definition of complexity they used was proposed by the panel, and was defined as “the more energy you spend to overview the picture of the mix, the more complex it is”. This definition shows some similarities to Kolmogorov complexity, or minimal description length. The Kolmogorov complexity of a string is defined as the minimal length of a programme on a universal Turing machine to obtain a string x [37].

The complexity of taste perception was studied in [27]. The authors looked into how the complexity of food products made it more difficult for sensory panels to assess the products. The products they investigated were mixtures of different tastes (sucrose, sodium chloride, citric acid, and caffeine) in water, iced tea, and tomato soup. They indicated that the definition of complexity involves more than just counting the number of constituents and taste sensations. The sensory sensation can change significantly when two or more components of qualitatively different stimuli are mixed. They can enhance, suppress, or mask one another, and react chemically, physiochemically, or biophysically. Thus, a linear relationship between the number of constituents and the perceived complexity of the product does not exist. Bitnes et al. also noted that by increasing the complexity of a product by adding a new stimulus dimension, such as texture, the product became more difficult to evaluate.

Giacalone et al. [13] based their definition of complexity on the one proposed by Berlyne [3], and defined complexity to be related to the number of discernible elements within a stimulus, and to the degree to which these elements coexist or conflict. They noted that complexity is closely related to perceived ambiguity of a stimulus, and to the cognitive effort necessary for its interpretation. Therefore, perceived complexity is not as straightforward as counting the number of compounds. We tend to perceive complex odours as unique stimuli, rather than as a muddle of components. Thus, the relationship between the chemical complexity of a mixture leading to a given flavour, as defined by Jellinek and Köster [15], and the perceived complexity of a flavour, is rapidly lost with an in increase in the number of flavour components.

Herrmann et al. [17] researched how scents influence cognition and spending on groceries. They developed scents varying in terms of complexity. They based this on the idea that more complex olfactory stimuli contain more information to be processed, thereby decreasing the ease of processing the information before deciding to buy, compared to simple scents. They exposed people to scents with different complexities and asked them to perform cognitive tasks, such as solving anagrams, during exposure to the scent. The amount of anagrams solved decreased with increasing complexity of the scent, indicating that more complex ambient scents decreased processing fluency. They found that simpler scents had more of an impact on consumers’ spending in retail situations.

## 3. Complexity in Sensory Science, Ecology, and Socio-Economics

The science of complexity crosses discipline boundaries, resulting in the reincarnation of measures of complexity in different guises within different contexts, which call for specific representations. For instance, Shannon information entropy has been proposed as a measure for biodiversity, in which context it is widely used and debated [38], along similar lines as in the behavioural sciences.

We note an interesting overlap between the discourse on the use of Shannon entropy in sensory science and biodiversity. In biodiversity, diversity is equated to uncertainty. The greater the uncertainty in predicting the outcome of a random sample from an ecosystem, the greater the diversity. Different measures of diversity have been proposed that bear different names, but most of them can be linked to each other using Shannon entropy. One of these measures is richness, which can just measure the number of species, or its equivalent number of selected attributes, or the number of ingredients in a recipe. A critique related to the measure of richness is that it does not take the distribution of species into account. This led to the introduction of measures such as Shannon entropy, linking diversity to the uncertainty of interpretation of entropy. The next level of critique is that two systems could have the same level of entropy, and thus complexity, but that these two systems could consist of a population with common species only, or a population consisting of common and rare species together. This critique bears similarities with the critique of Luce and Laming, in that all sensations are measured with equal weight, and therefore do not allow the incorporation of partial new experiences into the measure of complexity. These incorporations are nonetheless important for perception, as put forward by Berlyne in his contributions 2 and 3 to complexity. Of course, these incorporations should also be considered from the intuitive biologist’s perspective on diversity.

In order to explain complexities other than sensory and ecological ones, for example, regarding traffic jams, fractures, and the like, one can resort to ways of expanding on the use of the term “information”. As such, extending the concept of entropy, so-called Tsallis entropy was introduced [39]. Depending on an order parameter alpha, rare species will contribute more or less to the diversity measure compared to a so-called uniform case of alpha = 1, which resembles the special Shannon entropy case. The introduction of Tsallis entropy has been argued to allow for the introduction of more structural information of the system into the analysis, facilitating explanations for the existence of power laws in, for example, socio-economic systems or biological systems. However, a major criticism of Tsallis entropy is that it does not represent an extensive measure, which contrasts with the extensive character of the state function of entropy [40], contrasting with the according mathematical convenience in the expression for the entropy, based on information-theoretical reasoning. This criticism is further substantiated by the fact that one can also explain power laws without an extended expression for entropy, by including structural information, but by means of a structural fractality within biological [41] and socio-economic systems [42]. This fractal structure facilitates the transport of matter and information throughout the system with minimised effort. In these analyses, entropy remains an extensive function, and there is no need to introduce a fitting parameter within the entropy itself that makes the entropy non-extensive. In other words, the structure within the system should be taken into consideration in the analysis of the system, in a manner that is self-consistent with the main underlying basis of extensivity of entropy. It is noted that the elegancy of this fractality approach lies in the fact that the fractality of such structures guarantees an entropy production that is smooth over a vast range of length scales, without any sinks or holes (discontinuities in the entropy production), leading to efficiencies in, for example, catalysis reactions in fractal geometries [43], or the maintenance of a constant temperature in the nose of a reindeer [44].

At this point, we like to refer to the third complexity property of Berlyne. Elements that can be grouped into one unit, with the equivalent of a population of organisms grouped into one species, are considered to have lower diversity than elements that cannot be grouped into one unit, with the equivalent population of a different species. For example, a population of 10 dogs is considered to have less diversity than a population of three dogs, four finches, and four ferns. This means that one needs to take into account the relations between species, as well as their specific ecosystems. Similarly, one needs to take into account attributes in sensory science for describing more complex interactions. For both, there exist different measures, both theoretical and empirical, that can add weight to the probabilities. In a recent contribution [45], it was shown that adding a similarity matrix, the elements add weight to the probability of observing a particular species in an ecosystem. Moreover, the authors’ proposed measure for diversity has the property of having a linear relation with the number of species present or the richness, but is additionally corrected for species distribution, rareness, and similarity. This linearity is important for the interpretation of the according complexity measure and value comparison. It is noted that such linearity is at odds with the use of the entropy/information expression as introduced by Shannon.

## 4. Information Transfer During a Food Sensory Experiment

In food sensory science, experiments often contain samples that need to be tested by a panellist, who can either be a trained or untrained panellist. A typical experiment has at least two components, the sample and the panellist.

Regarding the sample part, we need to assume that any probability distribution of sample attributes remains the same during the experiment. This implies that the samples should be physically and chemically stable during the experiment. To produce a stable sample, and ensure that all samples are used throughout the experiment, a robust and well-described process is required. This information should be encoded in a recipe. We could say that the sample is a manifestation of the recipe which, in a poetic sense, contains the message of its creator. Elements from the process have found their way into complexity measures in food sensory science. Most frequently, this has been along the dimension of number of ingredients. In a typical recipe, the number of food ingredients is encoded by the mass fraction or volume fraction. The use of merely the number of ingredients is not without criticism, as already pointed out in Section 2.

Regarding the perception part of the experiment, similar considerations apply as for the sample part. Panellists are asked to recognise the number of ingredients or sensations. The frequency of occurrence of perception of ingredients versus the total number of ingredients could be a measure of complexity, as described with information theory; however, like for the sample part above, this is still sensitive to criticisms, e.g., as espoused by Luce [29] and Laming [30], and discussed in the introduction of Section 2. The mapping of the sample and of the recipe to the sensory perception output produced by the panellist is an acceptable analysis [30]. As a second problem, there is the required incorporation of the context, i.e., the information coming from the environment and circumstances outside of the panellist, and beyond the stimuli directly initiated by the food.

We conclude that we have probabilities of different sorts for the input (sample) and output (perception) of the experiment. These inputs and outputs allow for the use of the concept of mutual information. Importantly, for the perception part of the experiment, different panellists may use different alphabets to encode their sensory experiences, for example, to distinguish the connoisseur from the ignoramus. A connoisseur has a more sophisticated encoding apparatus, using a better-trained sensory network, which allows for better discrimination of recipes than is possible for the ignoramus. The latter usually encodes with fewer attributes, which are, moreover, less capable of distinguishing between recipes. The use of a larger vocabulary (of the connoisseur) means that a long string of information made by the connoisseur contains potentially more information than an equal length of string made by the ignoramus. Whether or not they are connoisseurs, overall, the training of panellists will influence the magnitude of any measure of complexity. The higher density of information of strings produced by connoisseurs likely makes the processing of the information slower. Thus, the time required for the processing of information, and the according energy use, is likely to be explicitly coupled with complexity [46].

Normalising alphabet size allows us to define redundancy in the amount of information, which can be compressed due to the underlying structure of the information. In this way, we can compare what the structure is like on the input side and on the output side.

The goal of the experiments is to relate a particular set of sample properties to perceived properties. Examples of perceived properties are arousal, satiety liking, and the sensation of a complex experience at the perceptual level. These form a mapping from one kind of property to another kind of property. Sometimes, these different kinds of properties cannot be related to each other, and can be considered independent. The concept of complexity is relevant to both sample and receiver properties. An example is the process of sample production and the actual sample properties, as in the sequence below:

recipe process -> sample properties -> *sensory process -> experience*

where the regular letter type refers to the sample part, and the italic letter type refers to the perceptual part. In most cases of sensory experiments, there are relations between the normal and italic types, but also among the normal type and among the italic type. Because not all kinds of properties can be related, one needs to select a set of properties that are deemed of interest, and form a hypothesis. If complexity is a relevant property, then a measure for it needs be constructed.

In this paper, we investigate the use of the concept of complexity and the property of complexity, in the context of sensory science. Information, or entropy, is often used as a measure for complexity in the sensory literature. In this way, we hope to provide some structure for the ongoing discourse of complexity in sensory data. However, complexity and information are not necessarily the same, as will become more clear below.

According to Berlyne [3,5], each individual strives for an optimal level of arousal (inverted U-shaped curve). The arousal potential of a stimulus is dependent on the degree of novelty, complexity, and intensity of the stimulus. The arousal potential indicates in which direction the hedonic value or liking of a product might change with repeated exposure. Products with a low arousal potential tend to show a decrease in liking, while products with a high arousal potential tend to show an increase in liking.

Sulmont-Rossé et al. [25] hypothesise that there may be two opposing processes taking place during repeated exposure to the same food. On the one hand, there is a decrease in uncertainty regarding the safety and identity of the food, while on the other hand, there is an increased feeling of boredom. A novel stimulus or product is more commonly associated with uncertainty and with conflict. These are both states that are more likely associated with negative than positive affect [47]. The introduction of a novel or complex stimulus poses a problem for an individual [48]. The individual has had limited prior experience with this low-probability stimulus. Therefore, they do not know how to respond to it. However, the stimulus does not leave the individual ambivalent, because it will have some resemblance to previously encountered stimuli. The individual has multiple possible responses to the stimulus. Many of these may be in competition. With increasing exposure to the stimulus, there is a reduction in response competition and tension. Too much exposure to the stimulus may again lead to boredom [49]. According to Sulmont-Rossé et al. [25], the balance between the effect of resolving uncertainty and the effect of boredom during repeated exposure may be dependent on the initial arousal potential of the product. They hypothesise that when someone is exposed to a stimulus with an arousal potential that is higher than their arousal optimum, the uncertainty-reducing effect may outweigh the boredom effect, and the appreciation of the individual for the stimulus will increase with exposure. To assess the arousal potential of soft drinks, Sulmont-Rossé et al. aggregated unfamiliarity (frequency of tasting, familiarity, and description), puzzlement (complexity and surprise), element (number of aromas and distinctiveness), and intensity into an index. They concluded that the more a drink was perceived as unfamiliar, the more the liking scores increased with exposure. They explained this by indicating that “new and complex stimuli may share some resemblance with stimuli encountered in the past and elicit antagonistic responses in the individual”. In this way, extra information is brought into the experiment, i.e., the information obtained in the past. This effect naturally adds to the complexity of the phenomenon of sensory perception. When there are no negative consequences associated with a new stimulus, the individual becomes less uncertain about the food, and the product may be regarded as safe. This reduces tension and increases food acceptance and liking.

As indicated, there are problems with the use of the term “complexity” in the literature. The term is ambiguous, and the definition varies from author to author. Because complexity and perceived complexity are so poorly defined, it is possible that individuals will allocate different meanings to the term “complexity”, not only based on their prior experiences, but also in relation to changing contexts [7]. The issue is complicated further because a stimulus may have different measures of complexity regarding each of its attributes. Some authors, such as Dember and Earl [50] and Smith and Dorfman [51], have proposed the use of other concepts such as variability, which can be defined in terms of a neutral metric derived from information theory. Complexity may be transformed into a psychological variable by defining it in terms of change in stimulation. Discrepancies/changes can arise during the observation of a stimulus. This indicates that there is uncertainty, or information, in the stimulus. In their study, Smith and Dorfman [51] used the Shannon measure of information, based on the works of Shannon [28] and Wiener [52], to classify their visual stimuli into different classes. Each class had the same level of uncertainty, measured in bits of information. Smith and Dorfman indicated that their information-theoretic concept of uncertainty was relatively unambiguous, contrary to complexity. This can be accommodated by using the premise that complexity is equal to information, and thus to the negative value of uncertainty, or entropy.

Adding further to the complexity of sensory perception of foods, one may combine different foods in a meal. When combining different foods, it may be of interest to describe the overall sensory impression of such combinations. Words such as harmony or complexity may be used for describing a meal or a dish, but it is sometimes difficult to relate specific sensory attributes to such descriptions. Such overall impressions can be described as composite or integrated attributes, meaning that they are composed of several sensory impressions, but that they are evaluated as a whole. Traditionally, in descriptive analysis, the aim is to use singular, non-redundant descriptors, with minimal overlap with other terms [53]. However, there have been several attempts with integrated approaches to describe overall impressions, such as total intensity of aroma and flavour, and the evaluation of balance or blend [54,55].

In this paper, we talk about stimulus complexity when it is related to the food product itself. In most cases, the number of ingredients is the key ingredient for a measure of complexity. As an example, according to Olabi et al. [11], more complex foods containing more spices were regarded as more flavourful and less bland. Some authors, however, challenge this view, due to the possibility of interaction between components, which has the potential to influence intensity or hedonistic values [8,26]. In addition to this point, it should be understood that when interactions between components are present, these add to the overall information of the system, and thus should be considered as a contributor to complexity.

In the context of perceived complexity, one can roughly discern two types of complexity measures: one based on the number of sensations that are selected (referred to as sensory complexity), and one that is measured on a scale (referred to as perceived complexity). An example of the first comes from Lévy et al. [12], who studied the complexity of orange juices to which they added different flavour extracts. They asked the panel to rate elaborateness, confusion, and homogeneity. They indicated that the perceived complexity was strongly correlated with the amount of selected flavours and novelty, but not much with the difficulty of describing the stimulus. However, it may be equally difficult to detect any one flavour. An example of perceived complexity is the use of the Visual Analogue Scale (VAS), or asking how difficult it is to describe a sensation.

Some publications combine two different forms of complexity. Weijzen et al. [18] studied the effects of intensity and complexity on sensory-specific satiety and food acceptance of various chicken soups and snacks, such as chocolate bars with incorporated nuts, with different flavours and textures. They assessed the complexity of the products by asking the panellists directly how complex the product was, how many different ingredients it contained, and how difficult it was to describe it. From their results, they concluded that complexity was strongly correlated with the number of perceived ingredients (sensory complexity), and that the difficulty of describing a product was inversely related to familiarity. They concluded that complexity decreased sensory-specific satiety. They also concluded that intensity increased sensory-specific satiety.

Ruijschop et al. [9] investigated the influence of complexity on satiation and food intake. Their samples consisted of two types of strawberry-aromatized sweetened yoghurts: one with a single-component strawberry aroma, and one with a multi-component strawberry aroma. They assessed the complexity by asking the panellists about the difficulty of describing the taste, the perceived number of ingredients (sensory complexity), and the perceived level of complexity. They observed significant differences in some of the complexity attributes (while the number of ingredients was not significantly different), but not in pleasantness. The multi-component aroma-flavoured yoghurt was able to enhance satiety because of increased sensory stimulation.

Paulsen et al. [32] indicated that complexity can be used to describe a meal or a dish, such as fish combined with a sauce, but that it is difficult to relate specific sensory attributes to such descriptions. They defined complexity as the sensation of many tastes and flavours.

As mentioned above, Lévy et al. [12] studied the complexity of orange juices to which they added different flavour extracts. Based on these attributes, they obtained the level of complexity for the different orange juices with added aromas and flavourings. They also asked the panellists about their initial liking for the products. The optimum level of complexity for each product and each panellist was obtained from the complexity level of the most-liked juice. Then, they exposed the panel to samples that were either more complex or less complex than their “complexity optimum”, and looked at how the optimum level of complexity changed with repeated exposure.

Soerensen et al. [10], while predicting long-term consumer liking of chocolate, defined perceived complexity as the number of flavours, amounting to more than 800 different ones. The release of flavours depends on eating behaviour, making flavour perception, and thus the complexity of the perception, a dynamic property. The input/output of information during an experiment on panellists’ perceptions while eating a food thus becomes dynamic. It is then impossible to talk about complexity. The annotation of perception complexity becomes even more difficult because of interpersonal differences in perception, and differences in previous experiences between panellists. Jellinek and Köster [15] defined this problem of many receptors being involved at different times during perception as physiological complexity.

What all of the above-described measures of complexity have in common is their relation with the number of perceived attributes. We note that the observed relation may not necessarily have the correct functional dependence, and may deviate from a linear one, as is the case while using the expression of entropy. The relation between complexity and the number of perceived attributes, and the according measure of complexity in terms of entropy, may be linked to the concept of richness of sensation, and it may bear similarity to the concept of species richness in ecology [38].

## 5. Complexity, Degree of Dissimilarity, and Degree of Structure

The complexity of texture perception was studied by Larsen et al. [33,34,35] and Marcano et al. [6]. As already mentioned in Section 2, Larsen et al. defined “textural complexity” as determined during mastication, from the first bite through to the point of swallowing [34]. The number of texture attributes at any one time was established by asking the panel to name all the attributes they perceived while consuming the sample. It was concluded that for more texturally complex samples, there were more changes in textural attributes. One could rephrase this by saying that the more dissimilar the perceptions were, the more complex the samples were perceived as being. This rephrasing brings complexity in tune with the concept of uncertainty and its quantification in terms of the entropy measure.

In another experimental setting with dissimilarity measurements, Marcano et al. [6] studied the texture perception of different cheese pies with added visible particles. Rating of the cheese pies in terms of complexity was performed on a nine-point scale, from not complex at all to very complex. It was found that high complexity coincided with high heterogeneity, i.e., a high level of dissimilarity.

Observing differences is one way of assessing complexity, as we saw in Section 5. Another way is detecting differences in patterns, revealing the structural form of the information. This was, for example, performed in the work by Mielby et al. [21,22], which we discussed earlier, where pictures of fruits and vegetables were mixed in different ratios and cut in different patterns.

Regarding the complexity of taste perception, as studied by Bitnes et al. [27], the complexity of food products was investigated by means of mixtures of different tastes (sucrose, sodium chloride, citric acid, and caffeine) in water, iced tea, and tomato soup. Complexity, like in other cases, involved more than just the number of constituents and taste sensations, and also contained mixed stimuli, making the structure of the perception more diverse. Adding a new stimulus dimension, such as texture, made the product become more difficult to evaluate, adding a new element to the information structure, and thus causing it to be perceived as more complex.

Giacalone et al. [13] related complexity to the number of discernible elements within a stimulus and to the degree to which these elements coexist or conflict, and found that complexity was closely related to perceived ambiguity of a stimulus, as well as to the cognitive effort necessary for its interpretation. Ambiguity can be viewed as the contribution to the entropy, i.e., the negative value of the information, and the effort to retrieve the information to come to an interpretation can be viewed as the amount of information required for this. In other words, these authors also found evidence for the relevance of the concept of entropy, and of information, to the concept of complexity.

Herrmann et al. [17] also considered the effort of obtaining information as a measure for complexity, as obtained in their study of how scents influence cognition. More complex olfactory stimuli contain more information to be processed, thereby requiring more effort. This was proven by the amount of anagrams the panellists were able to solve.

## 6. Discussion and Future Directions 

The measurement of complexity has been dealt with ambiguously in the sensory literature and beyond. Many studies show a relation between (perceived) complexity and the number of stimuli or ingredients. This is, likely correctly, debated, because interactions among the ingredients or stimuli also contribute to information, which is left out when only considering the number of ingredients. This is furthermore exemplified by means of a study by Ruijschop et al. [9], which showed that with a constant number of ingredients in products, the perceived complexity of these products still differed.

The most logical measure for complexity seems to be an information measure. Such a measure has also been used in some sensory food-related complexity studies. Notably, the functional dependency between information and number of ingredients is not a linear one, which constitutes another argument as to why complexity is most likely not related linearly to the number of ingredients in a food. The Shannon information measure, and its negative value, the uncertainty or entropy measure, may suffice for many occasions, although in this case, one needs to meticulously take into account the real number of possibilities of perceived signals, including those related to the context of the sensory experiment.

It is believed that re-analysing the appropriate sensory perception literature in terms of complexity as a Shannon information measure, instead of being simply equal to the number of ingredients or number of stimuli, will add to interpreting such sensory perception, and facilitate comparisons among different experiments. This was exemplified in reference [2].

Incorporating such a context-dependent element, however, poses challenges when using a measure of sensory profile complexity, particularly when complexity is defined as the effort required to retrieve the sensory profile. Such a measure relates back to the definition of complexity given by Kolmogorov, but seems hard to quantify unambiguously, due to the unclarity regarding how to embed context into the expression, which is in turn partially due to the fact that the measure will be dependent on the level of training of the individual that is perceiving the food.

Experiments probing dissimilarities can directly provide a measure of entropy, the negative value of the information, and as such, the negative value of complexity.

Overall, many experiments performed in the food sensory field point to the fact that complexity can be equated to the “amount of information obtained in the perception and interpretation process while consuming the food”, and can be quantified in terms of an entropy measure by following the Shannon entropy measure. This explains the different possible experimental ways to determine (differences in) complexity, because the entropy measure can be obtained from experiments determining differences in either ambiguity, uncertainty, dissimilarity, or the amount of effort needed to obtain the information. This holds for food sensory perception in particular, and complex systems in general.

It is believed that using the Shannon entropy measure may enable better comparison among sensory perception outcomes determined in different laboratories and in different experimental settings, and that such use may also uncover possible differences in the contexts of sensory perception experiments. At the same time, considering sensory perception as the output of the complex system of “a human consuming food”, and using the Shannon information to contribute towards its understanding, it is foreseeable that scientists who want to explore complex systems from a fundamental point of view may resort to the outcomes of food sensory perception experiments.

## Data Availability

No new data have been generated. All references used and referred to are publicly available.
